# N-acetyl-cysteine administration during foetal life improves social behaviour and restores hippocampal bdnf levels in adolescent mice prenatally exposed to a high-fat diet

**DOI:** 10.1192/j.eurpsy.2021.1220

**Published:** 2021-08-13

**Authors:** C. Musillo, A. Berry, B. Collacchi, E. Pieroni, M. Lepre, K. Creutzberg, V. Begni, M.A. Riva, F. Cirulli

**Affiliations:** 1 Center For Behavioral Sciences And Mental Health, Isituto Superiore di Sanità, Rome, Italy; 2 Department Of Psychology, Sapienza, University of Rome, Rome, Italy; 3 Department Of Pharmacological And Biomolecular Sciences, University of Milan, Milan, Italy

**Keywords:** foetal programming, social anxiety, N-acetyl-cysteine, maternal obesity

## Abstract

**Introduction:**

Maternal obesity may affect foetal programming representing a risk for adult mental health. Oxidative stress and inflammation associated with maternal obesity can alter the maturation of neuronal circuits affecting behaviour and mood.

**Objectives:**

We investigated the emotional phenotype of male and female mouse offspring born from a high-fat diet (HFD) fed dams. We also tested the efficacy of N-acetyl-cysteine (NAC – an antioxidant) in preventing the negative effects of HFD. We focused on adolescence, an age of main vulnerability for the onset of psychopathology.

**Methods:**

Female C57BL/6N mice were fed HFD for 13 weeks and, after 5 weeks, were also exposed to NAC (1 g/kg b.w.) via drinking water, until delivery. The neurodevelopment of offspring was assessed through the homing test. Emotionality was assessed in 35-45-day-old adolescent mice through elevated-plus-maze (EPM) and social interaction tests (SIT). Transcriptomic analysis of hippocampal tissue were performed to identify mechanisms of action of both HFD and NAC.

**Results:**

NAC was effective in moderating body weight gain in HFD-fed dams. Neither HFD or NAC affected offspring development. Regardless of sex, prenatal HFD reduced exploration and decreased sociability, in EPM and SIT respectively. Prenatal HFD decreased hippocampal levels of BDNF in female offspring. Prenatal NAC administration prevented social anxiety and restored BDNF levels in the HFD group.

**Conclusions:**

Data indicate long-term effects of maternal obesity on dams’ weight, offspring’s behaviour and hippocampal BDNF levels. These effects may be mediated by changes in oxidative stress as NAC was effective as a preventive agent. ERANET-NEURON-JTC 2018 (Mental Disorders) Project “EMBED”.

